# Supplementary material for: Efficient termination of nuclear lncRNA transcription promotes mitochondrial genome maintenance
Source: eLife. 2018 Mar 5;7:e31989. doi: 10.7554/eLife.31989 (PMC5837560; doi:10.7554/eLife.31989)
Supplement: Supplementary file 1. — Names, genotypes, purposes and origins of yeast strains used in the manuscript are indicated. [file elife-31989-supp1.docx]

**Supplementary file 1: Supplementary Table S1 Yeast strains**

| **Name** | **Identifier** | **Genotype** | **Used in Figure** | **Published** |
| --- | --- | --- | --- | --- |
| wild type | BY4741 | Mat a, ura3∆0, leu2∆0, his3∆1, met15∆0 | Fig.1,2,3,4, Fig1-S1, Fig2-S1 Fig3-S1, Fig4-S1 | (Winzeler et al., 1999) |
| BY4741 + V0 | SMY2020 | Mat a, ura3∆0, leu2∆0, his3∆1, met15∆0 + SMC339 | Fig.2 , Fig. 2-S1 | in this study |
| BY4741; rrp6∆ | rrp6∆ | Mat a, ura3∆0, leu2∆0, his3∆1, met15∆0, rrp6∆::KanMX | Fig.2, Fig2-S1, Fig3-S1 | (Winzeler et al., 1999) |
| BY4741; xrn1∆ | xrn1∆ | Mat a, ura3∆0, leu2∆0, his3∆1, met15∆0, xrn1∆::KanMX | Fig.2, Fig2-S1, Fig3-S1 | (Winzeler et al., 1999) |
| circuit I | SMY2481 | Mat a, ura3∆0, leu2∆0, his3∆1, met15∆0, ppt1∆::mCherry::NAT, sut129∆::YFP, ppt1/sut129pro∆::MED2/CUT60pro::CUT60 | Fig.4, Fig4-S1 | in this study |
| circuit II | SMY2484 | Mat a, ura3∆0, leu2∆0, his3∆1, met15∆0, ppt1∆::mCherry::NAT, sut129∆::YFP, ppt1/sut129pro∆::MED2/CUT60pro::SUT129 | Fig.4, Fig4-S1 | in this study |
| cut60∆::5'C-3'S, sut129∆::URA3 | SMY246 | Mat a, cut60∆::5'CUT60-3'SUT129, sut129∆::URA3, ura3∆0, leu2∆0, his3∆1, met15∆0 | Fig.3 , Fig3-S1 | in this study |
| cut60∆::5'C-3'S, sut129∆::URA3, rrp6∆ | SMY255 | Mat a, cut60∆::5'CUT60-3'SUT129, sut129∆::URA3, ura3∆0, leu2∆0, his3∆1, met15∆0, rrp6∆::KanMX | Fig3-S1 | in this study |
| cut60∆::5'C-3'S, sut129∆::URA3, xrn1∆ | SMY241 | Mat a, cut60∆::5'CUT60-3'SUT129, sut129∆::URA3, ura3∆0, leu2∆0, his3∆1, met15∆0, xrn1∆::KanMX | Fig3-S1 | in this study |
| cut60∆::5'S-3'C, sut129∆::URA3 | SMY240 | Mat a, cut60∆::5'SUT129-3'CUT60, sut129∆::URA3, ura3∆0, leu2∆0, his3∆1, met15∆0 | Fig.3, Fig3-S1 | in this study |
| cut60∆::5'S-3'C, sut129∆::URA3, rrp6∆ | SMY251 | Mat a, cut60∆::5'SUT129-3'CUT60, sut129∆::URA3, ura3∆0, leu2∆0, his3∆1, met15∆0, rrp6∆::KanMX | Fig3-S1 | in this study |
| cut60∆::5'S-3'C, sut129∆::URA3, xrn1∆ | SMY253 | Mat a, cut60∆::5'SUT129-3'CUT60, sut129∆::URA3, ura3∆0, leu2∆0, his3∆1, met15∆0, xrn1∆::KanMX | Fig3-S1 | in this study |
| cut60∆::CUT217 | SMY2720 | Mat a, cut60∆::CUT217, ura3∆0, leu2∆0, his3∆1, met15∆0 | Fig.3 | in this study |
| cut60∆::CUT48 | SMY2695 | Mat a, cut60∆::CUT48, ura3∆0, leu2∆0, his3∆1, met15∆0 | Fig.3 | in this study |
| cut60∆::CUT#78 | SMY2682 | Mat a, cut60∆::CUT#78, ura3∆0, leu2∆0, his3∆1, met15∆0 | Fig.3 | in this study |
| cut60∆::CUT170 | SMY2685 | Mat a, cut60∆::CUT170, ura3∆0, leu2∆0, his3∆1, met15∆0 | Fig.3 | in this study |
| cut60∆::CUT277 | SMY2109 | Mat a, cut60∆::CUT277, ura3∆0, leu2∆0, his3∆1, met15∆0 | Fig.3 | in this study |
| cut60∆::CUT60#1 | SMY2127 | Mat a, cut60∆::CUT60, ura3∆0, leu2∆0, his3∆1, met15∆0 | Fig.3 | in this study |
| cut60∆::CUT60#2 | SMY2128 | Mat a, cut60∆::CUT60, ura3∆0, leu2∆0, his3∆1, met15∆0 | Fig.2 | in this study |
| cut60∆::CUT95 | SMY2108 | Mat a, cut60∆::CUT95, ura3∆0, leu2∆0, his3∆1, met15∆0 | Fig.3 | in this study |
| cut60∆::SUT129, sut129∆::URA3 | SMY149 | Mat a, cut60∆::SUT129, sut129∆::URA3, ura3∆0, leu2∆0, his3∆1, met15∆0 | Fig.1, Fig.3, Fig3-S1 | in this study |
| cut60∆::SUT129, sut129∆::URA3; rrp6∆ | SMY267 | Mat a, cut60∆::SUT129, sut129∆::URA3, ura3∆0, leu2∆0, his3∆1, met15∆0, rrp6∆::KanMX | Fig.1 , Fig3-S1 | in this study |
| cut60∆::SUT129, sut129∆::URA3; xrn1∆ | SMY150 | Mat a, cut60∆::SUT129, sut129∆::URA3, ura3∆0, leu2∆0, his3∆1, met15∆0, xrn1∆::KanMX | Fig.1, Fig3-S1 | in this study |
| cut60∆::URA3 | SMY81 | Mat a, cut60∆::URA3, ura3∆0, leu2∆0, his3∆1, met15∆0 | Fig.1,2,3,4, Fig1-S1, Fig2-S1, Fig3-S1, Fig4-S1 | in this study |
| cut60∆::URA3#1 | SMY2154 | Mat a, cut60∆::URA3, ura3∆0, leu2∆0, his3∆1, met15∆0 | Fig.1-S1 | in this study |
| cut60∆::URA3#2 | SMY2155 | Mat a, cut60∆::URA3, ura3∆0, leu2∆0, his3∆1, met15∆0 | Fig.1-S1 | in this study |
| cut60∆::URA3#3 | SMY2156 | Mat a, cut60∆::URA3, ura3∆0, leu2∆0, his3∆1, met15∆0 | Fig.1-S1 | in this study |
| cut60∆::URA3, sut129∆::CUT60 | SMY117 | Mat a, cut60∆::URA3, sut129∆::CUT60, ura3∆0, leu2∆0, his3∆1, met15∆0 | Fig.2 | in this study |
| cut60∆::URA3, sut129∆::CUT60; rrp6∆ | SMY139 | Mat a, cut60∆::URA3, sut129∆::CUT60, ura3∆0, leu2∆0, his3∆1, met15∆0, rrp6∆::KanMX | Fig.2 | in this study |
| cut60∆::URA3, sut129∆::CUT60; xrn1∆ | SMY107 | Mat a, cut60∆::URA3, sut129∆::CUT60, ura3∆0, leu2∆0, his3∆1, met15∆0, xrn1∆::KanMX | Fig.2 | in this study |
| cut60∆::URA3; rrp6∆ | SMY260 | Mat a, cut60∆::URA3, ura3∆0, leu2∆0, his3∆1, met15∆0, rrp6∆::KanMX | Fig.2, Fig2-S1, Fig3-S1 | in this study |
| cut60∆::URA3; xrn1∆ | SMY84 | Mat a, cut60∆::URA3, ura3∆0, leu2∆0, his3∆1, met15∆0, xrn1∆::KanMX | Fig.2, Fig2-S1, Fig3-S1 | in this study |
| cut60∆::URA3++ | SMY2612 | Mat a, cut60∆::URA3++, ura3∆0, leu2∆0, his3∆1, met15∆0 | Fig.3, Fig.4, Fig4-S1 | in this study |
| cut60∆::URA3+ | SMY2610 | Mat a, cut60∆::URA3+, ura3∆0, leu2∆0, his3∆1, met15∆0 | Fig.3 | in this study |
| cut60∆::URA3+ trp1-terminator | SMY2717 | Mat a, cut60∆::URA3+trp1- terminator, ura3∆0, leu2∆0, his3∆1, met15∆0 | Fig.3 | in this study |
| mip1Δ | Deletion Library | Mat a, mip1∆::kanMX, ura3∆0, leu2∆0, his3∆1, met15∆0 | Fig.1, Fig1-S1, Fig.3 | (Winzeler et al., 1999) |
| nrd1∆151-214 | SMY2693 | Mat a, ura3∆0, leu2∆0, his3∆1, met15∆0,nrd1∆151-214 | Fig.3, Fig3-S1 | (Vasiljeva et al., 2008) |
| W303 | SMY2617 | Mat a, leu2-3,112, trp1-1, can1-100, ura3-1, ade2-1, his3-11,15 | Fig.3, Fig3-S1 | (Winzeler et al., 1999) |
| nrd1-1 | SMY2694 | MATa, ura3-52, leu2-3,112, trp1-1, his3-11,15, nrd1∆::HIS3, lys2∆2, ade2-1, met2∆1, can1-100 [pRS424nrd1-1[ts allele],TRP1,2µ ori] | Fig.3, Fig3-S1 | (Steinmetz and Brow, 1998) |
| set∆ in circuit I | SMY2495 | Mat a, set2∆::kanMX, ura3∆0, leu2∆0, his3∆1, met15∆0, ppt1∆::mCherry::NAT, sut129∆::YFP, ppt1/sut129pro∆::MED2/CUT60pro::CUT60 | Fig.4, Fig4-S1 | in this study |
| set2∆ in circuit II | SMY2522 | Mat a, set2∆::kanMX, ura3∆0, leu2∆0, his3∆1, met15∆0, ppt1∆::mCherry::NAT, sut129∆::YFP, ppt1/sut129pro∆::MED2/CUT60pro::SUT129 | Fig.4, Fig4-S1 | in this study |
| SMY81+ V0 | SMY2023 | Mat a, cut60∆::URA3, ura3∆0, leu2∆0, his3∆1, met15∆0 + SMC339 | Fig.2 , Fig. 2-S1, Fig. 2 | in this study |
| SMY81+ V1 | SMY2026 | Mat a, cut60∆::URA3, ura3∆0, leu2∆0, his3∆1, met15∆0 + SMC340 | Fig.2 , Fig. 2-S1, Fig. 2 | in this study |
| SMY81+ V2 | SMY2032 | Mat a, cut60∆::URA3, ura3∆0, leu2∆0, his3∆1, met15∆0 +SMC342 | Fig.2 , Fig. 2-S1, Fig. 2 | in this study |
| SMY81+ V3 | SMY2097 | Mat a, cut60∆::URA3, ura3∆0, leu2∆0, his3∆1, met15∆0 + SMC361 | Fig.2 , Fig. 2-S1, Fig. 2 | in this study |
| SMY81+ V4 | SMY2099 | Mat a, cut60∆::URA3, ura3∆0, leu2∆0, his3∆1, met15∆0 + SMC362 | Fig.2 , Fig. 2-S1 , Fig. 2 | in this study |
| SPT b/g | SMY921 | Mat x, ura3∆0, his3∆200, lys2-128∂ | Fig.4, Fig4-S1 | (Simchen et al., 1984) |
| SPT b/g in circuit I | SMY2504 | Mat x, ura3∆0, his3∆200, lys2-128∂, ppt1∆::mCherry::NAT, sut129∆::YFP, ppt1/sut129pro∆::MED2/CUT60pro::CUT60 | Fig.4, Fig4-S1 | in this study |
| SPT b/g in circuit II | SMY2534 | Mat x, ura3∆0, his3∆200, lys2-128∂,ppt1∆::mCherry::NAT, sut129∆::YFP, ppt1/sut129pro∆::MED2/CUT60pro::SUT129 | Fig.4, Fig4-S1 | in this study |
| spt16∆ in circuit I | SMY2507 | Mat x, ura3-52, his4-912∂, lys2-128∂, spt16-197, ppt1∆::mCherry::NAT, sut129∆::YFP, ppt1/sut129pro∆::MED2/CUT60pro::CUT60 | Fig.4, Fig4-S1 | in this study |
| spt16∆ in circuit II | SMY2516 | Mat x, ura3-52, his4-912∂, lys2-128∂, spt16-197, ppt1∆::mCherry::NAT, sut129∆::YFP, ppt1/sut129pro∆::MED2/CUT60pro::SUT129 | Fig.4, Fig4-S1 | in this study |
| spt6∆ in circuit II | SMY2510 | Mat x, ura3-52, leu2∆1, his3∆200, lys2-128∂, FLAG-spt6-1004,ppt1∆::mCherry::NAT, sut129∆::YFP, ppt1/sut129pro∆::MED2/CUT60pro::SUT129 | Fig.4, Fig4-S1 | in this study |
| spt6∆ in circuit I | SMY2519 | Mat x, ura3-52, leu2∆1, his3∆200, lys2-128∂, FLAG-spt6-1004, ppt1∆::mCherry::NAT, sut129∆::YFP, ppt1/sut129pro∆::MED2/CUT60pro::CUT60 | Fig.4, Fig4-S1 | in this study |
| sut129∆::URA3 | SMY93 | Mat a, sut129∆::URA3, ura3∆0, leu2∆0, his3∆1, met15∆0 | Fig.2 , Fig1-S2 | in this study |
| sut129∆::URA3; rrp6∆ | SMY257 | Mat a, sut129∆::URA3, ura3∆0, leu2∆0, his3∆1, met15∆0, rrp6∆::KanMX | Fig.2 | in this study |
| sut129∆::URA3; xrn1∆ | SMY89 | Mat a, sut129∆::URA3, ura3∆0, leu2∆0, his3∆1, met15∆0, xrn1∆::KanMX | Fig.2 | in this study |
