# Supplementary material for: Efficient termination of nuclear lncRNA transcription promotes mitochondrial genome maintenance
Source: eLife. 2018 Mar 5;7:e31989. doi: 10.7554/eLife.31989 (PMC5837560; doi:10.7554/eLife.31989)
Supplement: Supplementary file 2. — Names, DNA sequences and purposes of oligonucleotides used in the manuscript are indicated. [file elife-31989-supp2.docx]

**Supplementary file 2: Supplementary Table S2 Oligonucleotides**

| **Identifier** | **Sequence** | **Note** |
| --- | --- | --- |
| MLO1328 | TAAAAGGTACCAATTCATTAAATATTATAATATTTACTTCTTACTAGTAATGAATTAAGTCTTGATATATAAC | Primers are used to insert CUT#78 into CUT60 locus, to generate SMY2682 (cut60∆::CUT#78) on SMC486 template. |
| MLO1329 | CTAGACGACTCCATAATGCGCTTTGATTGGCTGGGCATCTATTTTCGTTACGGTATCCTCACCATGCCA | Primers are used to insert CUT#78 into CUT60 locus, to generate SMY2682 (cut60∆::CUT#78) on SMC486 template. |
| MLO1334 | CTAGACGACTCCATAATGCGCTTTGATTGGCTGGGCATCTATTTTCGTTActccttacgcatctgtgc | Primers are used to generate PCR fragment to make SMY2717. |
| MLO1335 | Gagaagatgcggccagcaaaacgttattactgagtagtatttatttaagtattg | Primers are used to generate PCR fragment to make SMY2717. |
| MLO1368 | Caatacttaaataaatactactcagtaataacgttttgctggccgcatcttctc | Primers are used to generate PCR fragment to make SMY2717. |
| MLO1376 | GCACAACTAAAAGGTACCAATTCATTAAATATTATAATATTTACTTCTTATTTGCGGTATGTTAATAAAGTTACC | Primers are used to insert CUT48 into CUT60 locus, to generate SMY2695 (cut60∆::CUT48). |
| MLO1377 | CTAGACGACTCCATAATGCGCTTTGATTGGCTGGGCATCTATTTTCGTTAGAATCAGTAATCACATCAACGAGATTC | Primers are used to insert CUT48 into CUT60 locus, to generate SMY2695 (cut60∆::CUT48). |
| MLO1370 | GCACAACTAAAAGGTACCAATTCATTAAATATTATAATATTTACTTCTTATAGAGTATATGCATTTCGGTAGTG | Primers are used to insert CUT217 into CUT60 locus, to generate SMY2720 (cut60∆::CUT217). |
| MLO1371 | CTAGACGACTCCATAATGCGCTTTGATTGGCTGGGCATCTATTTTCGTTAATTGACAAATCATATTATATAGTGAAAAGCG | Primers are used to insert CUT217 into CUT60 locus, to generate SMY2720 (cut60∆::CUT217). |
| MLO431 | CTGCAGGAATTCGATATCAAGCTTCTTTAACGAAAATAGATGCCCAGCCA | Primers are used to generate fragment for SMC340, and for sequencing of SMC340. |
| MLO433 | CTGCAGGAATTCGATATCAAGCTTCTATTACACGAAAGACAAGTGTGAAATG | Primers are used to generate fragment for SMC342, and for sequencing of SMC342. |
| MLO434 | CGAGGTCGACGGTATCGATAAGCTTTAGGGCTTTCATACAGGATTTAAAGAAACA | Primers are used to generate fragment for SMC340, SMC342, SMC361, SMC362 and for sequencing of SMC340, SMC342, SMC361, SMC362. |
| MLO466 | TCATTGAATTTCGTCGCTAAG | Primers are used to generate PCR fragment for ATP16 Northern probe. |
| MLO467 | AGTTACACATAACTGCGAGTCTGGT | Primers are used to generate SMY2154-2156 (cut60∆::URA3#1-3), and to amplify ATP16 Northern probe. |
| MLO486 | CTGCAGGAATTCGATATCAAGCTTTACTTTCAGACGGGCAGTTTATT | Primers are used to generate fragment for SMC361, and for sequencing of SMC361. |
| MLO487 | CTGCAGGAATTCGATATCAAGCTTGCACAACAGATAATGTAACGGTC | Primers are used to generate fragment for SMC362, and for sequencing of SMC362. |
| MLO488 | TCCTAATCATTGTAATTAAGACGTT | Primers are used to generate PCR product to make SMY2127, SMY2128 (cut60∆::CUT60), and genotyping all strains where CUT60 has been replaced with another sequence. |
| MLO489 | CATCTAATTTAACATGAACTTTTACAAT | Primers are used to generate PCR product to make SMY2127, SMY2128 (cut60∆::CUT60), and genotyping all strains where CUT60 has been replaced with another sequence. |
| MLO496 | CAACTAAAAGGTACCAATTCATTAAATATTATAATATTTACTTCTTACTAAATATTTTGACGCATCGAACTTTAT | Primers are used to generate PCR product to make SMY2108 (cut60∆::CUT95). |
| MLO497 | CTAGACGACTCCATAATGCGCTTTGATTGGCTGGGCATCTATTTTCGTTAATATAATGGCCTGCTGTTCCTAAAA | Primers are used to generate PCR product to make SMY2108 (cut60∆::CUT95). |
| MLO498 | CAACTAAAAGGTACCAATTCATTAAATATTATAATATTTACTTCTTACTAATCTTCCTACAATTCATTTTGTCCT | Primers are used to generate PCR product to make SMY2109 (cut60∆::CUT277). |
| MLO499 | CTAGACGACTCCATAATGCGCTTTGATTGGCTGGGCATCTATTTTCGTTAGGACATGAAGCATGAGAGATAATCA | Primers are used to generate PCR product to make SMY2109 (cut60∆::CUT277). |
| MLO502 | CTATATATTGAAGCCGCTGAGGTCTAAT | Primers are used for sequencing SMC362. |
| MLO503 | ATGGTAGTACAAAATAGCCCAGTTTCGT | Primers are used for sequencing SMC362. |
| MLO515 | ACGAAACTGGGCTATTTTGTACTACCAT | Primers are used for sequencing of SMY2127 (cut60∆::CUT60), and to generate SMY2154-2156 (cut60∆::URA3#1-3). |
| MLO516 | CTTGATGCACTCTTTCCAATAATTGAACGT | Primers are used for sequencing of SMY2127 (cut60∆::CUT60) and genotyping cut60∆::URA3#1-3. |
| MLO517 | AAATCATAAGAAATTCGCTTATTTAGA | Primers are used to generate SMC 425 and SMC 426. |
| MLO1319 | AAAAGGTACCAATTCATTAAATATTATAATATTTACTTCTTACTAGTTTGACTTGGAAAATTTTAGATGC | Primers are used to insert CUT170 into CUT60 locus, to generate SMY2285 (cut60∆::CUT170). |
| MLO1320 | CGACTCCATAATGCGCTTTGATTGGCTGGGCATCTATTTTCGTTAAAGTAGGTAGCTGTGGAAAGCAGCT | Primers are used to insert CUT170 into CUT60 locus, to generate SMY2285 (cut60∆::CUT170). |
| MLO776 | CCCTTGCTCACCATTTTGTTATACTTTCAGACGGGCAGTTTATT | Primers are used to generate SMC 425 and SMC 426. |
| MLO777 | TAGCCGTGAGGATAGGAAATAACAAAATGTCTAAAGGTGAAGAAT | Primers are used to generate SMC 425 and SMC 426. |
| MLO778 | ATTCTTCACCTTTAGACATTTTGTTATTTCCTATCCTCACGGCTA | Primers are used to generate SMC 425 and SMC 426. |
| MLO779 | AATAAACTGCCCGTCTGAAAGTATAACAAAATGGTGAGCAAGGG | Primers are used to generate SMC 425 and SMC 426. |
| MLO843 | CCACCCGTAGGTATTGAAGC | Primers are used for qPCR to test for mtDNA presence and to generate COX3 Northern probe. |
| MLO844 | CTGCGATTAAGGCATGATGA | Primers are used for qPCR to test for mtDNA presence and to generate COX3 Northern probe. |
| MLO845 | GCTGATGTTATTCATGATTTTGC | Primers are used for qPCR to test for mtDNA presence. |
| MLO846 | CATGACCTGTCCCACACAAC | Primers are used for qPCR to test for mtDNA presence. |
| MLO847 | TGCCACAATTAGTTCCATTTT | Primers are used for qPCR to test for mtDNA presence. |
| MLO488 | GATCATAGGTAAAAAGAATTGTGAGAA | Primers are used for qPCR to test for mtDNA presence. |
| MLO933 | CGTATGCGATGCACAACTAAAAGGTACCAATTCATTAAATATTATAATATTTACTTCTTAAGGAAACGAAGATAAATCATGTCGA | Primers are used to generate PCR fragment to make SMY2478 and SMY2717. |
| MLO934 | CCATAATGCGCTTTGATTGGCTGGGCATCTATTTTCGTTAcagggtaataactgatataattaaattgaa | Primers are used to generate PCR fragment to make SMY2610. |
| MLO935 | CGACTCCATAATGCGCTTTGATTGGCTGGGCATCTATTTTCGTTATTCTTTAATAGTGGACTCTTGTTCC | Primers are used to generate PCR fragment to make SMY2612. |
| SB1055 | GCAGAATTCACAGAAAACACTTCGAGAT | Primers are used to generate rrp6Δ::KanMX. |
| SB1056 | GAGCAAGCTTCTTTTCTAACTTGGAAGT | Primers are used to generate rrp6Δ::KanMX. |
| MLO1182 | CATTATGGAGTCGTCTAGGAAG | Primers are used for ChIP-qPCR, promoter ATP16. |
| MLO1185 | CAGTGTGTGTACGATACCTTTAT | Primers are used for ChIP-qPCR, promoter ATP16. |
| MLO1186 | AGGTGTCGTTGAAGTTATGGAAG | Primers are used for ChIP-qPCR, ATP16 gene body. |
| MLO1187 | CACATAACTGCGAGTCTGGTTG | Primers are used for ChIP-qPCR, ATP16 gene body. |
| MLO1249 | TGGATTCCGGTGATGGTGTT | Primers are used for ChIP-qPCR. Used in Marquardt et al. 2014. |
| MLO1250 | AAATGGCGTGAGGTAGAGAGAAAC | Primers are used for ChIP-qPCR. Used in Marquardt et al. 2014. |
| SB2471 | TACCAAGGCAAGCGTTATTTC | Primers are used to generate sut129∆::URA3. |
| SB2570 | AAGTCGACTCGCTCTCTGCCCACAGCAGGAAGGA | Primers are used to generate sut129∆::URA3. |
| SB2801 | ACGGAAAAAGAAATACATACATACAATGATTGAAAAAATATTTTCATAAAGAAGAACTATTACACGAAAGACAAGTGTGAAATG | Primers are used to generate sut129∆::CUT60. |
| SB2802 | AGGAAATAAGAGTGCTACAAAAAATGACGATTACTTCTTTTTTCGTTCTAGTGATTACAGGCGGTGACCGAGGTGAAT | Primers are used to generate sut129∆::CUT60. |
| SB2803 | CGTATGCGATGCACAACTAAAAGGTACCAATTCATTAAATATTATAATATTTACTTCTTAAGGAAACGAAGATAAATCATGTCGA | Primers are used to generate cut60∆::URA3. |
| SB2804 | GACGGCCCCTTCCTAGACGACTCCATAATGCGCTTTGATTGGCTGGGCATCTATTTTCGTTAAAGTTAGTTTTGCTGGCCGCATCTTCT | Primers are used to generate cut60∆::URA3. |
| SB2875 | ACTAAAAGGTACCAATTCATTAAATATTATAATATTTACTTCTTAAATGAGAAGGAACTTTTGAGG | Primers are used to generate cut60∆::5'S-3'C and cut60∆::SUT129. |
| SB2876 | TCCATAATGCGCTTTGATTGGCTGGGCATCTATTTTCGTTAAAGTGATATCGCCGCTCATTGTTCTAT | Primers are used to generate cut60∆::5'C-3'S and cut60∆::SUT129. |
| SB2877 | ACAAACAAGAAGAGGTTAGAAAGCA | Primers are used to generate xrn1Δ::KanMX. |
| SB2878 | TGTAAAATCGAATTCATATCCAGGT | Primers are used to generate xrn1Δ::KanMX. |
| SB2898 | CAACTTCAAATAATATAGAAAAGAAAAATTTTATGATAAAAAAATACAACATG | Primers are used to generate cut60∆::5'C-3'S. |
| SB2899 | CATGTTGTATTTTTTTATCATAAAATTTTTCTTTTCTATATTATTTGAAGTTG | Primers are used to generate cut60∆::5'C-3'S. |
| SB2900 | GTGGTATTAGATTTGTCATGGTGTAAGAAGACTATAAGCTAAAAATGTAGAC | Primers are used to generate cut60∆::5'S-3'C. |
| SB2901 | GTCTACATTTTTAGCTTATAGTCTTCTTACACCATGACAAATCTAATACCAC | Primers are used to generate cut60∆::5'S-3'C. |
| SB2902 | TGCACTCTTTCCAATAATTGAACGTAACAT | Primers are used to genotype cut60∆::5'S-3'C and cut60∆::5'C-3'S. |
| SB2823 | TACTTTCAGACGGGCAGTTTATTTG | Primers are used to generate cut60∆::5'C-3'S. |
| SB2453 | TGTGGAACCCCAACAATTATC | Primers are used to amplify SUT129 Northern probe. |
| SB2454 | ATCGCCGCTCATTGTTCTAT | Primers are used to amplify SUT129 Northern probe. |
| SB3080 | ACAGTGGGAACGACAATAACAATAC | Primers are used to amplify MED2 Northern probe. |
| SB3081 | CGGCTGGAGGAGGGTTATC | Primers are used to amplify MED2 Northern probe. |
| SB2451 | AGCTCTGGCCTTGCAATAAA | Primers are used to amplify ATP16 promoter Northern probe. |
| SB2452 | CCCCTTCCTAGACGACTCCA | Primers are used to amplify ATP16 promoter Northern probe. |
| SB1777 | CAAACTTGTGTGCTTCATTG | Primers are used to amplify URA3 Northern probe. |
| SB1778 | TGTAACGTTCACCCTCTACC | Primers are used to amplify URA3 Northern probe. |
