# Supplementary material for: Efficient termination of nuclear lncRNA transcription promotes mitochondrial genome maintenance
Source: eLife. 2018 Mar 5;7:e31989. doi: 10.7554/eLife.31989 (PMC5837560; doi:10.7554/eLife.31989)
Supplement: Supplementary file 3. — Names, identifiers and purposes of plasmids used in the manuscript are indicated. [file elife-31989-supp3.docx]

**Supplementary file 3: Supplementary Table S3 Plasmids**

| **Name** | **Identifier** | **Note** |
| --- | --- | --- |
| V0 | SMC339 | Plasmid V0 used as a negative control and used in SMY2023. Plasmid selection in yeast using LEU. |
| V1 | SMC340 | Plasmid V1 is made by including sequence from promoter of ATP16 to ATP16 terminator into V0 and used in SMY2026. Plasmid selection in yeast using LEU. |
| V2 | SMC342 | Plasmid V2 is made by including sequence from CUT60 transcription start to ATP16 terminator into V0 and used in SMY2032. Plasmid selection in yeast using LEU. |
| V3 | SMC361 | Plasmid V3 is made by including sequence from promoter of CUT60 to ATP16 terminator into V0 and used in SMY2097. Plasmid selection in yeast using LEU. |
| V4 | SMC362 | Plasmid V4 is made by including sequence from terminator of MED2 to ATP16 terminator into V0 and used in SMY2099. Plasmid selection in yeast using LEU. |
| pRS316 | SMC88 | Plasmid is used to amplify URA3++ PCR fragment to use in SMY2478. |
| circuit I | SMC425 | Plasmid used in SMY2484, SMY2522, SMY2528, SMY2531, SMY2534, SMY2510 and SMY2516. |
| circuit II | SMC426 | Plasmid used in SMY2495, SMY2525, SMY2498, SMY2504, SMY2501, SMY2519 and SMY2507. |
| #78-plasmid | SMC486 | Plasmid used as DNA template to generate SMY2682. |
| SMC147 | pRS314 | Plasmid used as DNA template to generate SMY2717. |
| SMC425/426 parent | SMC50 | Plasmid used to make SMC425 and SMC426, SMC50 is published in Marquardt et al. 2014. |
